# Supplementary material for: The relationship between the source of oral health information and dental caries: Findings from Child Dental Health Survey 2013 in England
Source: PLoS One. 2024 Jul 1;19(7):e0302488. doi: 10.1371/journal.pone.0302488 (PMC11216584; doi:10.1371/journal.pone.0302488)
Supplement: Attachment — Submitted filename: FINAL PONE-D-23-31617 (1).pdf [file pone.0302488.s001.pdf]

PLOS ONE

The relationship between the source of oral health information and dental caries: Findings from Child Dental Health Survey 2013 in England

--Manuscript Draft--

**Manuscript Number:**	PONE-D-23-31617
**Article Type:**	Research Article
**Full Title:**	The relationship between the source of oral health information and dental caries: Findings from Child Dental Health Survey 2013 in England
**Short Title:**	The relationship between the source of oral health information and dental caries
**Corresponding Author:**	Ahmad Sofi-Mahmudi, DDS<br>McMaster University<br>Hamilton, Ontario CANADA
**Keywords:**	oral health; Health Literacy; Internet Use; Parents; Television
**Abstract:**	Objective: To determine the magnitude and shape of the relationship between dental caries experience and the source of oral health information in England.<br>Methods: This was a cross-sectional study using the Child Dental Health Survey 2013 in England. Using a negative binomial model, the relationship between the number of decayed, missing, filled teeth (DMFT) of 12- and 15-year-old students and their primary source of oral health information was assessed. The sources of oral health information included parents, TV, newspapers, the Internet, and social media. The adjusted model included age, sex, and the Index of Multiple Deprivation (IMD). R was used for data handling, analysis and reporting.<br>Results: Overall, 2,372 children were assessed (48.7% female, 48.6% 12-year-old). For the majority, the primary source of oral health information was their parents (89.5%) followed by the Internet (43.4%). Over nine-tenth of the participants had a DMFT=0. The adjusted model showed that the prevalence rate of DMFT for the children whose primary source of information is their parents (0.45) or TV (0.62) is lower than 1. The prevalence rate for the Internet (1.17) and social media (1.67) was higher than 1, but they were removed from the final model due to being non-statistically significant. Age and deprivation had a direct relationship with the prevalence rate of DMFT, meaning that 15-year-olds and children from more deprived areas had a higher prevalence rate of DMFT.<br>Conclusion: Children whose primary source of oral health information was their parents or TV had a lower DMFT. On the contrary, using the Internet or social media as the source of oral health information was associated with higher caries experience among schoolchildren.
**Order of Authors:**	Ahmad Sofi-Mahmudi, DDS
**Additional Information:**	
**Question**	**Response**
**Financial Disclosure**<br><br>Enter a financial disclosure statement that describes the sources of funding for the work included in this submission. Review the submission guidelines for detailed requirements. View published research articles from *PLOS ONE* for specific examples.<br><br>This statement is required for submission and **will appear in the published article** if	The author(s) received no specific funding for this work.

Powered by Editorial Manager® and ProduXion Manager® from Aries Systems Corporation

the submission is accepted. Please make sure it is accurate.<br><br>**Funded studies**<br>Enter a statement with the following details:<br>• Initials of the authors who received each award<br>• Grant numbers awarded to each author<br>• The full name of each funder<br>• URL of each funder website<br>• Did the sponsors or funders play any role in the study design, data collection and analysis, decision to publish, or preparation of the manuscript?<br><br>Did you receive funding for this work?	
**Competing Interests**<br><br>Use the instructions below to enter a competing interest statement for this submission. On behalf of all authors, disclose any competing interests that could be perceived to bias this work—acknowledging all financial support and any other relevant financial or non-financial competing interests.<br><br>This statement is required for submission and **will appear in the published article** if the submission is accepted. Please make sure it is accurate and that any funding sources listed in your Funding Information later in the submission form are also declared in your Financial Disclosure statement.<br><br>View published research articles from *PLOS ONE* for specific examples.	The authors have declared that no competing interests exist.

**NO authors have competing interests**<br><br>Enter: *The authors have declared that no competing interests exist.*<br><br>**Authors with competing interests**<br><br>Enter competing interest details beginning with this statement:<br><br>*I have read the journal's policy and the authors of this manuscript have the following competing interests: [insert competing interests here]*<br><br>* typeset	
**Ethics Statement**<br><br>Enter an ethics statement for this submission. This statement is required if the study involved:<br><br>• Human participants<br>• Human specimens or tissue<br>• Vertebrate animals or cephalopods<br>• Vertebrate embryos or tissues<br>• Field research<br><br>Write "N/A" if the submission does not require an ethics statement.<br><br>General guidance is provided below. Consult the submission guidelines for detailed instructions. **Make sure that all information entered here is included in the Methods section of the manuscript.**	The Research Ethics Committee at University College London granted approval for the CDHS 2013 (Project ID: 2000/003).

Powered by Editorial Manager® and ProduXion Manager® from Aries Systems Corporation

**Format for specific study types**<br><br>**Human Subject Research (involving human participants and/or tissue)**<br>• Give the name of the institutional review board or ethics committee that approved the study<br>• Include the approval number and/or a statement indicating approval of this research<br>• Indicate the form of consent obtained (written/oral) or the reason that consent was not obtained (e.g. the data were analyzed anonymously)<br><br>**Animal Research (involving vertebrate animals, embryos or tissues)**<br>• Provide the name of the Institutional Animal Care and Use Committee (IACUC) or other relevant ethics board that reviewed the study protocol, and indicate whether they approved this research or granted a formal waiver of ethical approval<br>• Include an approval number if one was obtained<br>• If the study involved *non-human primates*, add *additional details* about animal welfare and steps taken to ameliorate suffering<br>• If anesthesia, euthanasia, or any kind of animal sacrifice is part of the study, include briefly which substances and/or methods were applied<br><br>**Field Research**<br><br>Include the following details if this study involves the collection of plant, animal, or other materials from a natural setting:<br>• Field permit number<br>• Name of the institution or relevant body that granted permission	
**Data Availability**<br><br>Authors are required to make all data underlying the findings described fully available, without restriction, and from the time of publication. PLOS allows rare exceptions to address legal and ethical concerns. See the PLOS Data Policy and FAQ for detailed information.	Yes - all data are fully available without restriction

A Data Availability Statement describing where the data can be found is required at submission. Your answers to this question constitute the Data Availability Statement and **will be published in the article**, if accepted.<br><br>**Important:** Stating 'data available on request from the author' is not sufficient. If your data are only available upon request, select 'No' for the first question and explain your exceptional situation in the text box.<br><br>Do the authors confirm that all data underlying the findings described in their manuscript are fully available without restriction?	
**Describe where the data may be found in full sentences. If you are copying our sample text, replace any instances of XXX with the appropriate details.**<br><br>• If the data are **held or will be held in a public repository**, include URLs, accession numbers or DOIs. If this information will only be available after acceptance, indicate this by ticking the box below. For example: *All XXX files are available from the XXX database (accession number(s) XXX, XXX.*).<br>• If the data are all contained **within the manuscript and/or Supporting Information files**, enter the following: *All relevant data are within the manuscript and its Supporting Information files.*<br>• If neither of these applies but you are able to provide **details of access elsewhere**, with or without limitations, please do so. For example:<br><br>*Data cannot be shared publicly because of [XXX]. Data are available from the XXX Institutional Data Access / Ethics Committee (contact via XXX) for researchers who meet the criteria for access to confidential data.*<br><br>*The data underlying the results presented in the study are available from (include the name of the third party*	All metadata and codes are available from https://osf.io/2vazm/.

and contact information or URL).<br>• This text is appropriate if the data are owned by a third party and authors do not have permission to share the data.<br><br>* typeset	
Additional data availability information:	Tick here if the URLs/accession numbers/DOIs will be available only after acceptance of the manuscript for publication so that we can ensure their inclusion before publication.

Powered by Editorial Manager® and ProduXion Manager® from Aries Systems Corporation

Manuscript Click here to access/download;Manuscript;manuscript_oral_health_source_up

The relationship between the source of oral health information and dental caries: Findings from Child Dental Health Survey 2013 in England

Ahmad Sofi-Mahmudi*[1,2], ORCiD: 0000-0001-6829-0823

1 National Pain Centre, Department of Anesthesia, McMaster University, Hamilton, ON, Canada.

2 Department of Health Research Methods, Evidence and Impact, McMaster University, Hamilton, ON, Canada.

Corresponding author: Ahmad Sofi-Mahmudi; **Address**: MDCL 2109, McMaster University, 1280 Main Street West, Hamilton, ON L8S 4K1, Canada; **Telephone**: +1 (905) 525-9140 ext. 22743; **Email**: sofima@mcmaster.ca, a.sofimahmudi@gmail.com.

Abstract

Objective: To determine the magnitude and shape of the relationship between dental caries experience and the source of oral health information in England.

Methods: This was a cross-sectional study using the Child Dental Health Survey 2013 in England. Using a negative binomial model, the relationship between the number of decayed, missing, filled teeth (DMFT) of 12- and 15-year-old students and their primary source of oral health information was assessed. The sources of oral health information included parents, TV, newspapers, the Internet, and social media. The adjusted model included age, sex, and the Index of Multiple Deprivation (IMD). R was used for data handling, analysis and reporting.

Results: Overall, 2,372 children were assessed (48.7% female, 48.6% 12-year-old). For the majority, the primary source of oral health information was their parents (89.5%) followed by the Internet (43.4%). Over nine-tenth of the participants had a DMFT=0. The adjusted model showed that the prevalence rate of DMFT for the children whose primary source of information is their parents (0.45) or TV (0.62) is lower than 1. The prevalence rate for the Internet (1.17) and social media (1.67) was higher than 1, but they were removed from the final model due to being non-statistically significant. Age and deprivation had a direct relationship with the prevalence rate of DMFT, meaning that 15-year-olds and children from more deprived areas had a higher prevalence rate of DMFT.

Conclusion: Children whose primary source of oral health information was their parents or TV had a lower DMFT. On the contrary, using the Internet or social media as the source of oral health information was associated with higher caries experience among schoolchildren.

Keywords: Oral Health, Health Literacy, Internet Use, Parents, Television

Introduction

Despite being largely preventable, dental caries persists as a substantial global health concern (1). The 2022 Global Oral Health Status Report by the World Health Organization highlights that more than two billion individuals worldwide currently experience untreated dental caries in their permanent teeth (2). In terms of economic impact, dental conditions resulted in both direct and indirect expenditures amounting to $544.41 billion in 2015, positioning it as the sixth most costly health condition on a global scale (3).

Effective communication and education about oral health may be crucial in preventing dental caries, particularly among children (4). Such oral health education plans can be about oral hygiene or the detrimental effects of poor dietary choices, tobacco use, and excessive alcohol consumption on oral health. Various sources, such as dental professionals, schools, parents, and media, play pivotal roles in disseminating oral health information (5). By empowering individuals with this knowledge, oral health education not only helps prevent dental problems but also encourages early intervention and treatment, ultimately contributing to improved overall oral health and well-being.

Previous research has focused on the effect of a specific source of information on oral health. However, the question of which one of these sources plays a more important role has remained unanswered. Therefore, the aim of this research article is to determine the relationship between the sources of oral health information and the number of decayed, missing, filled teeth (DMFT) among 12- and 15-year-old children living in England in 2013.

Methods

All codes and metadata related to the study were shared via its OSF repository (https://osf.io/2vazm/) at the time of submission of the manuscript.

Is this a prerequisite in this journal and must it be included?

Source of the data

Rephrase- Study Design this was a retrospective cross sectional study using the CDHS 2023 data....

I used the Child Dental Health Survey (CDHS) 2013 dataset to answer the research question. The CDHS 2013 represents the fifth installment within the sequence of decennial cross-sectional national surveys conducted in the United Kingdom, focusing on children's health. The study encompassed students aged 5, 8, 12, and 15 who were enrolled in mainstream state and independent schools across England, Wales, and Northern Ireland. The survey encompassed a total of 13,628 students. Data acquisition for the CDHS 2013 involved multiple methods, including a clinical dental examination, a self-completion questionnaire administered to older children, and a parental questionnaire. For younger children, parental consent was obtained through a written opt-in process, while older children followed an opt-out procedure. The Research Ethics Committee at University College London granted approval for the CDHS 2013 (Project ID: 2000/003). Additional comprehensive information regarding the survey's design has been previously published elsewhere (6).

Possible sources of information in this dataset include parents, TV, newspapers and magazines, the internet, and social media. Permanent teeth caries experience (DMFT) was considered as the dependent variable; therefore, I only included 12- and 15-year-olds. Age, sex, and deprivation level (based on country-specific Indices of Multiple Deprivation or IMD) were used for model adjustment.

Data analysis

As DMFT is count data, regression models with count data distributions were chosen. Among them, Poisson regression is the most widely used. However, since the data showed over-dispersion, a negative binomial regression was used. Overdispersion was confirmed by comparing the mean (0.19) and standard deviation (0.79) of DMFT. Also, the result of the overdispersion test showed an overdispersion estimate of 1.95 (p<0.001).

Results

Descriptive statistics

This dataset included 2,372 children from England. Of these, 51.3% were male, and 48.7% were female. DMFT (outcome of interest) was a highly skewed variable with many zeros; therefore, both the median and interquartile range were 0. In Table 1, frequencies and weight-adjusted proportions are reported for age groups, IMD, and sources of information.

Dont start a sentence with abbreviation rather use "The DMFT" remove outcome of interest- you are writing for a scientific audience so this should be obvious to them.

Table 1. Descriptive statistics of included variables

**Characteristic**	**Number (%)**
Sex	
Female	1222 (48.7)
Male	1150 (51.3)
Age	
12 years	1215 (48.6)
15 years	1157 (51.4)
Index of Multiple Deprivation (England)	
Most deprived quintile	986 (32.5)
2nd quintile	448 (20.1)

Characteristic	Number (%)
3rd quintile	305 (14.4)
4th quintile	340 (18.2)
Least deprived quintile	293 (14.9)
Source of oral health information	
Parents	2140 (89.5)
Internet	1042 (43.4)
TV programs	852 (35.5)
Newspapers or magazines	580 (26.2)
Social media	351 (14.3)
DMFT	
0	2071 (90.2)
1	183 (6.1)
More than 1	118 (3.7)

Hypothesis testing

Eight different models were constructed: five for the unadjusted relationship between DMFT and each source of oral health information; one with all sources; one with all sources and adjusted for sociodemographic variables; and the final model. Table 2 depicts the summary for each model. I should note that the numbers for categorical variables are (prevalence) rate ratios. All numbers in Table 2 are back-transformed from their logarithm provided by the statistical software. Here, we scrutinize models 6-8.

Table 2. The results of different regression models

Model	Intercept	Parents	TV	Newspapers	Internet	Soc Media	Age 15	Female	IMD 5th	IMD 4th	IMD 3rd	IMD 2nd

**1**	0.35 (0.23-0.56) ***	0.47 (0.29-0.75) **	—	—	—	—	—	—	—	—	—	—
**2**	0.20 (0.17-0.25) ***	—	0.76 (0.54, 1.07)	—	—	—	—	—	—	—	—	—
**3**	0.19 (0.16-0.23) ***	—	—	0.87 (0.61, 1.27)	—	—	—	—	—	—	—	—
**4**	0.17 (0.14-0.22) ***	—	—	—	1.17 (0.84, 1.61)	—	—	—	—	—	—	—
**5**	0.17 (0.14-0.20) ***	—	—	—	—	1.67 (1.10, 2.61) *	—	—	—	—	—	—
**6**	0.34 (0.22-0.55) ***	0.46 (0.28-0.73) **	0.74 (0.52-1.05) *	0.90 (0.62-1.32)	1.18 (0.82-1.69)	1.68 (1.07-2.68) *	—	—	—	—	—	—
**7**	0.51 (0.30-0.92) *	0.44 (0.26-0.70) ***	0.59 (0.41-0.85) **	0.87 (0.60-1.28)	1.20 (0.83-1.73)	1.52 (0.97-2.41)	1.43 (1.04-1.98) *	1.13 (0.82-1.56)	0.19 (0.10-0.33) ***	0.17 (0.10-0.30) ***	0.64 (0.40-1.03)	0.61 (0.40-0.93) *
**8 (final)**	0.59 (0.36-1.01)	0.45 (0.27-0.72) ***	0.62 (0.44-0.88) **	—	—	—	1.47 (1.06-2.03) *	—	0.17 (0.09-0.30) ***	0.17 (0.10-0.29) ***	0.59 (0.37-0.95) *	0.63 (0.42-0.96) *

*: P<0.05; **: P<0.01; ***: P<0.001

The value of the intercept in the negative binomial model stands for the expected (average) value of the count dependent variable when the values of all predictor variables in the dataset are set to 0. Here, the expected value of DMFT when all other variables are 0 is expected to be in the range of 0.17–0.59.

All the following numbers are from adjusted models. Therefore, they are the estimates for the degree of association between each variable and DMFT, holding all other variables in the model constant.

When all sources of oral health information and sociodemographic variables were added in model 7, two sources (internet and social media) and two demographic variables (being of age 15 and female) had a direct relationship with DMFT. It means that schoolchildren using these sources, girls, and those aged 15, had more tooth decay experience (higher prevalence rate ratio). However, three sources, newspapers and magazines, the internet and social media, and sex, did not have a significant relationship with DMFT. Therefore, they were excluded from the final model.

The final model includes parents and TV as sources of oral health information, as well as age and deprivation as sociodemographic variables. This model shows that the expected DMFT for

schoolchildren receiving information from their parents was 0.45 times the expected DMFT for schoolchildren who did not receive such information from their parents. Also, schoolchildren who used a TV as a source had a 38% lower rate of DMFT on average than those who did not use a TV.

Fifteen-year-olds had a 47% higher prevalence rate of DMFT compared with 12-year-olds. Looking at IMD quintiles (categorical variable) reveals a gradient that schoolchildren from less deprived backgrounds had a lower rate of DMFT on average compared to those from the most deprived category. For instance, the two least deprived categories (IMD 5th and 4th) had 83% lower prevalence rates of DMFT compared with the most deprived category (IMD 1st, the reference group).

The final model formula is as follows:

$$DMFT = 0.59 + 0.45X_{parents} + 0.62X_{TV} + 1.47X_{age} + 0.17X_{JMD5} + 0.17X_{JMD4} + 0.59X_{JMD3} + 0.63X_{JMD2} + \varepsilon$$

Testing model fitness

When comparing the final model with model 7 (including all variables) using the likelihood ratio test, the associated chi-squared value was -6.56 (df=4, p=1). Therefore, there is no evidence that the final model underperforms compared with the model with all the variables.

Checking model assumptions

Negative binomial models assume the conditional means are not equal to the conditional variances. This inequality is captured by estimating a dispersion parameter that is held constant in a Poisson model. Thus, the Poisson model is actually nested in the negative binomial model (2). I used a likelihood ratio test to compare these two and test this model assumption. The associated chi-

squared value was 626.56 with 4 degrees of freedom ($p<0.001$). This strongly suggests the negative binomial model, estimating the dispersion parameter, is more appropriate than the Poisson model.

Discussion

This study showed a decrease in the prevalence rate of DMFT in students whose sources for oral health information were their parents (0.45) or TV (0.62). Fifteen-year-olds and students from deprived areas had a higher prevalence rate. The prevalence rate for newspaper was lower than 1, and for the Internet and social media, were higher than 1; however, these were not statistically significant.

The lower prevalence rate for parents can be linked to socioeconomic status, as parents who are more vigilant about their children’s oral health and provide guidance tend to experience less deprivation (7,8). Higher socioeconomic status affords parents improved access to education and oral health information, making them more aware of the significance of proper oral hygiene and dental care practices (9). Consequently, these parents often impart their knowledge to their children, emphasizing the importance of regular dental check-ups, correct brushing techniques, and a healthful diet.

Moreover, socioeconomic status can significantly shape dietary choices within households. Families with higher incomes tend to enjoy greater access to nutritious foods and snacks, thereby contributing to enhanced oral health outcomes (10). Conversely, lower-income families may find themselves relying more on processed foods and sugary snacks, recognized culprits in dental decay.

In this study, an intriguing pattern emerged regarding the sources of oral health information among children. Those who relied on TV as their primary source were less likely to experience dental caries, while the Internet and social media were associated with a higher prevalence rate of dental caries (DMFT). This divergence in outcomes can be attributed to several factors related to the nature and oversight of these information sources.

Television, as a medium, offers better controllability when compared to the Internet and social media. TV programs operate within a centralized framework and typically undergo more rigorous quality checks before content is broadcasted. Consequently, the information presented on TV tends to be curated, reliable, and accurate, contributing to better dental health outcomes.

Furthermore, the role of parental involvement cannot be understated. Parents often monitor and guide their children's TV viewing habits, ensuring that they are exposed to educational content related to oral health (11). This proactive engagement by parents serves to reinforce the oral health messages conveyed through TV, encouraging children to adopt and practice good oral hygiene habits.

In contrast, the Internet and social media represent decentralized systems where anyone can post information, much of which may be inaccurate or misleading (12). Unfortunately, children's use of these platforms is not always subject to parental supervision or guidance. This lack of oversight can result in children accessing unreliable sources of information and potentially adopting less effective oral health practices. For example, children might be influenced by friends or online influencers who engage in behaviours like excessive sugary food consumption or neglecting oral hygiene.

Limitations

Because of the excessive number of zeros in the DMFT variable in this dataset, it is suggested to use a zero-inflated negative binomial regression (3). Zero DMFT may be because of different processes, such as receiving fluoridated water. A standard negative binomial model does not distinguish between processes that lead to zeros, but a zero-inflated model allows for and accommodates this complication. The same as above was done here for comparing the final model with a zero-inflated one. The associated chi-squared value was 2.51 (df=4, p=0.643). Therefore, using a non-zero-inflated model is preferable due to its simplicity.

Conclusion

Children whose primary source of oral health information was their parents or TV had a lower DMFT. On the contrary, using the Internet or social media as the source of oral health information was associated with higher caries experience among schoolchildren.

References

1. Peres MA, Macpherson LMD, Weyant RJ, Daly B, Venturelli R, Mathur MR, et al. Oral diseases: a global public health challenge. Lancet Lond Engl. 2019 Jul 20;394(10194):249–60.
2. World Health Organization. Global oral health status report: towards universal health coverage for oral health by 2030 [Internet]. Geneva: World Health Organization; 2022. 100 p. Available from: https://www.who.int/publications/i/item/9789240061484
3. Righolt AJ, Jevdjevic M, Marcenes W, Listl S. Global-, Regional-, and Country-Level Economic Impacts of Dental Diseases in 2015. J Dent Res. 2018 May;97(5):501–7.
4. Gurav KM, Shetty V, Vinay V, Bhor K, Jain C, Divekar P. Effectiveness of Oral Health Educational Methods among School Children Aged 5–16 Years in Improving their Oral Health Status: A Meta-analysis. Int J Clin Pediatr Dent. 2022 Jun 30;15(3):338–49.
5. Sistani MMN, Yazdani R, Virtanen J, Pakdaman A, Murtomaa H. Oral health literacy and

information sources among adults in Tehran, Iran. Community Dent Health. 2013 Sep;30(3):178–82.
6. Anderson T, Thomas C, Ryan R, Dennes M, Fuller E. Children's dental health survey 2013. Health Soc Care Inf Cent Engl. 2015;
7. Chen L, Hong J, Xiong D, Zhang L, Li Y, Huang S, et al. Are parents' education levels associated with either their oral health knowledge or their children's oral health behaviors? A survey of 8446 families in Wuhan. BMC Oral Health. 2020 Dec;20(1):203.
8. Brega AG, Jiang L, Johnson RL, Wilson AR, Schmiege SJ, Albino J. Health Literacy and Parental Oral Health Knowledge, Beliefs, Behavior, and Status Among Parents of American Indian Newborns. J Racial Ethn Health Disparities. 2020 Aug;7(4):598–608.
9. Park JB, Han K, Park YG, Ko Y. Association between socioeconomic status and oral health behaviors: The 2008–2010 Korea national health and nutrition examination survey. Exp Ther Med. 2016 Oct;12(4):2657–64.
10. Alkerwi A, Vernier C, Sauvageot N, Crichton GE, Elias MF. Demographic and socioeconomic disparity in nutrition: application of a novel Correlated Component Regression approach. BMJ Open. 2015 May 11;5(5):e006814–e006814.
11. Sormunen M, Turunen H, Tossavainen K. Self-reported bedtimes, television-viewing habits and parental restrictions among Finnish schoolchildren (aged 10–11 years, and 2 years later aged 12–13 years): Perspectives for health. Eur J Commun. 2016 Jun;31(3):283–98.
12. Lazer DM, Baum MA, Benkler Y, Berinsky AJ, Greenhill KM, Menczer F, et al. The science of fake news. Science. 2018;359(6380):1094–6.
