# Supplementary material for: The relationship between the source of oral health information and dental caries: Findings from Child Dental Health Survey 2013 in England
Source: PLoS One. 2024 Jul 1;19(7):e0302488. doi: 10.1371/journal.pone.0302488 (PMC11216584; doi:10.1371/journal.pone.0302488)
Supplement: Attachment — Submitted filename: Article review Plos one NOV 2023.docx [file pone.0302488.s002.docx]

Article review Plos one: The relationship between the source of oral health information and dental caries: Findings from Child Dental Health Survey 2013 in England

Title and Abstract:

- Gives a brief about what the article is about, significance of the study while the aim is stated clearly.

Introduction :

- Scientific background and rational stated
- Objectives not clearly stated

Methods:

- Study design not stated.
- Setting or data source described.
- Rationale for selecting the age groups (participants) is not explained.
- Method of sampling not clearly described.
- Sample size not mentioned, it is only mentioned in the results.
- No explain of how the study size was arrived at.
- Statistical methods are well described.

Discussion:

- No clear discussion of the implications of the results to the scientific field
- The authors did not clearly provide reasoned arguments for their interpretation of their results.
- No comparison with previous studies ,no highlight of similarities or differences.

General comments:

- Grammatical errors, need editing of the article.
- Write using third person.
- Data Zip file was empty
