# Supplementary material for: The relationship between the source of oral health information and dental caries: Findings from Child Dental Health Survey 2013 in England
Source: PLoS One. 2024 Jul 1;19(7):e0302488. doi: 10.1371/journal.pone.0302488 (PMC11216584; doi:10.1371/journal.pone.0302488)
Supplement: Attachment — Submitted filename: Response to reviewers_PLOS ONE_rev1.docx [file pone.0302488.s003.docx]

Reviewer #1: The author needs to describe the findings in the results as much as the description of the models in the results section, ensure that table 1 is in one page. The research ethics were followed promptly as this is a secondary data analysis from a primary survey data source. The author has to elaborate on that in a scientific way.

Authors: Thanks for your comment. We amended the manuscript accordingly.

Reviewer #2:

Title and Abstract:

- Gives a brief about what the article is about, significance of the study while the aim is stated clearly.

Authors: Thanks for your comment.

Introduction :

- Scientific background and rational stated
- Objectives not clearly stated

Authors: Thanks for your comment. We revised the objectives.

Methods:

- Study design not stated.

Authors: Thanks for your comment. We added the study design.

- Setting or data source described.
- Rationale for selecting the age groups (participants) is not explained.

Authors: Thanks for your comment. We added the rationale for selecting these age groups. These age groups were the only age groups with the data.

- Method of sampling not clearly described.

Authors: Thanks for your comment. We added the sampling method.

- Sample size not mentioned, it is only mentioned in the results.

Authors: Thanks for your comment. We already mentioned sample size under the “Sources of the data” in the Methods section.

- No explain of how the study size was arrived at.

Authors: Thanks for your comment. We added the details about the sample size.

- Statistical methods are well described.

Discussion:

- No clear discussion of the implications of the results to the scientific field

Authors: Thanks for your comment. We added more implications to the Discussion.

- The authors did not clearly provide reasoned arguments for their interpretation of their results.

Authors: Thanks for your comment. We already had provided the arguments for our interpretations (e.g., the 2^nd^ and 3^rd^ paragraph of the Discussion).

- No comparison with previous studies ,no highlight of similarities or differences.

Authors: Thanks for your comment. We added a paragraph in this regard.

General comments:

- Grammatical errors, need editing of the article.
- Write using third person.
- Data Zip file was empty

Authors: Thanks for your comment. We checked all the errors and amended the manuscript accordingly.
